# Therapeutic response in the HAWK and HARRIER trials using deep learning in retinal fluid volume and compartment analysis

**DOI:** 10.1038/s41433-022-02077-4

**Published:** 2022-05-06

**Authors:** Ursula Schmidt-Erfurth, Zufar Mulyukov, Bianca S. Gerendas, Gregor S. Reiter, Daniel Lorand, Georges Weissgerber, Hrvoje Bogunović

**Affiliations:** 1 Laboratory for Ophthalmic Image Analysis, Department of Ophthalmology and Optometry, Medical University of Vienna, Vienna, Austria; 2 Novartis Pharma AG, Basel, Switzerland

**Keywords:** Retinal diseases, Macular degeneration

## Abstract

**Objectives:**

To assess the therapeutic response to brolucizumab and aflibercept by deep learning/OCT-based analysis of macular fluid volumes in neovascular age-related macular degeneration.

**Methods:**

In this post-hoc analysis of two phase III, randomised, multi-centre studies (HAWK/HARRIER), 1078 and 739 treatment-naive eyes receiving brolucizumab or aflibercept according to protocol-specified criteria in HAWK and HARRIER, respectively, were included. Macular fluid on 41,840 OCT scans was localised and quantified using a validated deep learning-based algorithm. Volumes of intraretinal fluid (IRF), subretinal fluid (SRF), pigment epithelial detachment (PED) for all central macular areas (1, 3 and 6 mm) in nanolitres (nL) and best corrected visual acuity (BCVA) change in ETDRS letters were associated using mixed models for repeated measures.

**Results:**

Baseline IRF volumes decreased by >92% following the first intravitreal injection and consistently remained low during follow-up. Baseline SRF volumes decreased by >74% following the first injection, while PED volume resolved by 68–79% of its baseline volume. Resolution of SRF and PED was dependent on the substance and regimen used. Larger residual post-loading IRF, SRF and PED volumes were all independently associated with progressive vision loss during maintenance, where the differences in mean BCVA change between high and low fluid volume subgroups for IRF, SRF and PED were 3.4 letters (*p* < 0.0001), 1.7 letters (*p* < 0.001) and 2.5 letters (*p* < 0.0001), respectively.

**Conclusions:**

Deep-learning methods allow an accurate assessment of substance and regimen efficacy. Irrespectively, all fluid compartments were found to be important markers of disease activity and were relevant for visual outcomes.

## Introduction

The neovascular type of age-related macular degeneration (nAMD) is recognised as one of the most aggressive ocular diseases with rapid progression and extensive morphological alteration associated with irreversible functional loss. Intravitreal administration of antibodies inhibiting vascular endothelial growth factor (VEGF), has introduced a paradigm-shift in the treatment of nAMD, substantially improving the prognosis, and has rapidly become the gold standard in the management of the disease [1]. Yet, despite superior visual outcomes in multiple prospective clinical trials, the real-world benefit remains consistently below the expectations of physicians and patients, and has largely remained below results from registration trials [2–4]. Large-scale real-world analyses reflect the limitations of the current practice of retreatment regimens with the three established anti-VEGF agents bevacizumab, ranibizumab and aflibercept, thus demonstrating the need for increased efficiency in the management of nAMD in routine clinical practice.

Among novel pharmacological developments, the humanised, single-chain variable fragment brolucizumab pursues the promise of enhanced efficacy by better target-tissue penetration and superior therapeutic durability [5]. In the OSPREY study, brolucizumab-treated patients subsequently underwent an extension of the dosing interval to every 12 weeks (q12w), thus providing the rationale for the design of HAWK and HARRIER trials [6], in which brolucizumab and aflibercept were compared in 1817 patients with active macular neovascularisation (MNV) due to AMD. Brolucizumab was proven non-inferior to aflibercept in respect to best corrected visual acuity (BCVA) at week 48 with >50% of brolucizumab 6 mg-treated eyes maintained on q12w dosing intervals. Anatomic outcomes favoured brolucizumab over aflibercept in respect to central retinal thickness (CRT) and presence/absence of fluid [7]. Based on these confirmatory registration trials brolucizumab was approved for clinical use and became available to treating ophthalmologists [8].

Although used as a measure in many trials, CRT is only a rough indicator for fluid recurrence or resolution, originating from the era of time-domain optical coherence tomography (OCT) with only six radial scans. However, spectral-domain OCT (SD-OCT) offers high-resolution raster scanning of the macular area visualising neurosensory layers and pathological biomarkers such as fluid in precise detail [9]. To reliably identify, localise and quantify macular fluid volume on SD-OCT, methods of advanced artificial intelligence (AI) have recently been introduced [10]. Deep learning offers automated algorithms providing an objective, repeatable and fast assessment of the response to anti-VEGF therapy reflecting dosing and regimen [11]. The therapeutic pattern is thereby represented by characteristic dynamics of different fluid compartments such as intraretinal (IRF) and subretinal fluid (SRF) and/or pigment-epithelial detachment (PED).

In this paper, we performed a comprehensive volumetric retinal fluid analysis of the large HAWK and HARRIER trial data set including a total of 41,840 SD-OCT scans. Advanced AI tools provided a detailed quantitative assessment of the therapeutic response in a fully automated, objective and reproducible manner. Most importantly, we investigated the association of fluid volumes in the different compartments with visual outcomes over the maintenance phase to objectively provide evidence about one of the most controversially discussed topics in AMD management: Does fluid volume and location matter and how should the community optimise the management of AMD patients now and in the future when reliable AI tools will be available.

## Materials and methods

### Participants and imaging protocols

AI-based analysis was performed on the SD-OCT scans of 1078 and 739 patients enrolled in the HAWK and HARRIER clinical trials (ClinicalTrials.gov identifiers: NCT02307682 and NCT02434328, respectively). HAWK and HARRIER were two 96-weeks, prospective, randomised, double-masked, phase 3 multi-centre studies to evaluate the efficacy and safety of intravitreal brolucizumab vs. aflibercept in treatment-naïve patients with neovascular AMD. Patients were randomised 1:1(:1) to receive brolucizumab 6 mg or aflibercept 2 mg (or brolucizumab 3 mg in HAWK only). Both studies adhered to the tenets of the declaration of Helsinki and Good Clinical Practice and all participants provided written informed consent prior to study inclusion. The two core studies were approved by independent Ethics Committees by the sponsor.

Aflibercept patients were treated with a conventional q8w regimen throughout the study duration. Brolucizumab patients were treated by q12w, but adjusted to q8w as early as week 16, if disease activity was diagnosed by the masked investigator and supported by protocol guidance based on functional and anatomical characteristics. The maintenance phase in this specific protocol was defined as the post-loading phase i.e. weeks 12–96. Disease activity assessments were conducted by the masked investigator at week 16, week 20 and every 12 weeks thereafter to determine the subsequent dosing interval. There were additional disease activity assessment visits in the HARRIER trial. The trial protocol is illustrated in Fig. S1.

Volumetric macula-centred OCT scans were acquired following a standardised imaging schedule using either (1) a Cirrus HD-OCT III device (Carl Zeiss Meditec, Dublin, CA, US) consisting of 512 × 128 × 1024 voxels with a size of 11.7 × 47.2 × 2.0 µm^3^, comprising a volume of 6 × 6 × 2 mm^3^, (2) a Spectralis device (Heidelberg Engineering, Heidelberg, Germany) consisting of 512 × 49 × 496 voxels with a size of 11.7 × 125.3 × 3.9 µm^3^, comprising a volume of 6 × 6.1 × 1.9 mm^3^, or (3) the Topcon T-1000, T-2000, Atlantis DRI (Topcon, Japan) consisting of 512 × 128 × 885 voxels with a size of 11.7 × 46.9 × 2.6 µm^3^ and a volume of 6 × 6 × 2.3 mm^3^.

BCVA was measured and OCT acquired monthly at all visits. The OCT scans of all patients were collected centrally by two reading centres (Vienna Reading Center and Duke Reading Center) after certification of study photographers according to a predefined imaging protocol. All OCT images were transferred post-hoc to the Laboratory for Ophthalmic Image Analysis (OPTIMA) at the Medical University of Vienna in a pseudonymised format to conduct the AI-based fluid analysis. The post-hoc analysis presented here was conducted in compliance with the Declaration of Helsinki and approval was obtained by the Ethics Committee at the Medical University of Vienna (EK Nr: 1246/2018).

### Automated fluid localisation and quantification by deep learning

 A fully automated three-dimensional OCT segmentation of the three fluid compartments was performed on all of the 41,840 (24,369 and 17,471 OCT scans from HAWK and HARRIER, respectively) available volumetric scans, using a previously developed and validated deep learning algorithm for IRF and SRF quantification, the Vienna Fluid Monitor (RetInSight, Vienna, Austria) [10]. The algorithm was demonstrated capable of providing precise quantification of the therapeutic fluid response by application in other clinical trial data sets and real-world data sets [11, 12]. A convolutional neural network identifies fluid on a pixel-level of an entire 3D volumetric SD-OCT scan. Every voxel was classified into one of the four classes: background, retina, IRF or SRF. PED was identified by segmenting the region between the retinal pigment epithelium (RPE) and Bruch’s membrane, which was delineated using an automated graph-theoretic method, part of the Iowa Reference Algorithms [13, 14]. Any segmented region was considered as PED, if it had a height >200 µm, or alternatively, a width >400 µm, as originally defined by professional reading centres (Vienna, Wisconsin, Duke) [15, 16]. Absolute volume quantities expressed in nanolitres (nL) (1 nL = 0.001 mm^3^) of IRF, SRF, and PED were computed within the central 1 mm, 3 mm and 6 mm macular fields.

### Statistical analysis

To test for differences in fluid volumes across the treatment arms and patient subgroups, a mixed model for repeated measures (MMRM) was applied on the full analysis set (FAS). To test for differences across treatment arms, the fixed factors were: baseline volume, age group (<75, ≥75 years), treatment, visit, and treatment-visit interaction. To test for differences across OCT devices, the fixed factors were: baseline volume, age group, device, visit, and device-visit interaction. To test for differences across racial subgroups, the fixed factors were: baseline volume, age group, race, visit, and race-visit interaction. Finally, to test for differences across age subgroups, the fixed factors were: baseline volume, age group, visit and age group-visit interaction. Fluid volumes were log(x + 1) transformed to adjust for the skewed non-normal distribution of fluid volumes. The results were back transformed for graphical representation on the original nL scale.

To evaluate a treatment agnostic association of BCVA and the amount of the residual post-loading dose fluid, *High* fluid volume subgroup was defined for each fluid type (IRF, SRF, PED) as patients with the highest 25% quartile of mean post-loading (weeks 12–96) fluid volumes in the central 6 mm. The remaining 75% of patients were classified as *Low* fluid volume subgroup. Because majority of eyes were dry by the end of the loading dose, an alternative subgroup definition based on the median volume would have utilised cutoff values of <1 nl for IRF and SRF, which would not be representative of *High* fluid volume subgroup. MMRM was then applied on FAS to model the change of BCVA from week 12, with the following fixed factors: BCVA at the week 12 visit, IRF, SRF, PED volume subgroups and all visit and volume subgroups interactions. The two studies, HAWK and HARRIER, were analysed separately to allow for replication of the findings.

## Results

Automated volume quantification provided measurements for 1076 of 1078 patients (23,980 of 24,369 OCT volumetric scans) in HAWK, and 736 of 739 (17,215 of 17,471 volumetric OCT scans) in HARRIER. This efficacy represents 99.7% of all patients and 98.5% of all OCT scans. A breakdown of the processed OCT scans per device vendor is shown in Table S1, which yields the following share of scans per each device: Spectralis 67%, Cirrus 29%, and Topcon 4%. Baseline fluid volume characteristics for each study and macular field are reported in Table 1.

**Table 1:** Baseline arithmetic mean and standard deviation (SD), together with median and interquartile range (IQR) of untransformed fluid volumes in nanolitres (nL) for each study, fluid compartment, and central area (macular field).

Study	Fluid	Area	Mean [nL]	SD [nL]	Median [nL]	IQR (nL)
HAWK	IRF	1 mm	21.88	39.29	1.45	26.59
HAWK	IRF	3 mm	70.71	126.40	9.08	84.83
HAWK	IRF	6 mm	89.28	160.84	12.46	109.40
HAWK	SRF	1 mm	17.55	37.04	0.61	17.24
HAWK	SRF	3 mm	112.75	190.74	33.34	137.35
HAWK	SRF	6 mm	315.34	456.24	133.99	419.89
HAWK	PED	1 mm	68.92	88.27	36.13	89.37
HAWK	PED	3 mm	310.92	439.85	158.27	343.44
HAWK	PED	6 mm	393.09	598.48	188.16	413.58
HARRIER	IRF	1 mm	19.58	40.57	0.57	20.22
HARRIER	IRF	3 mm	62.09	121.52	4.44	67.47
HARRIER	IRF	6 mm	81.96	167.68	7.17	87.09
HARRIER	SRF	1 mm	20.17	35.86	1.95	26.41
HARRIER	SRF	3 mm	123.35	177.29	53.43	159.52
HARRIER	SRF	6 mm	364.32	498.97	177.38	455.51
HARRIER	PED	1 mm	69.23	91.77	36.13	89.30
HARRIER	PED	3 mm	337.50	452.38	179.12	359.68
HARRIER	PED	6 mm	425.40	583.12	227.12	451.09

### Response of retinal fluid to therapy

**Fig. 1:**
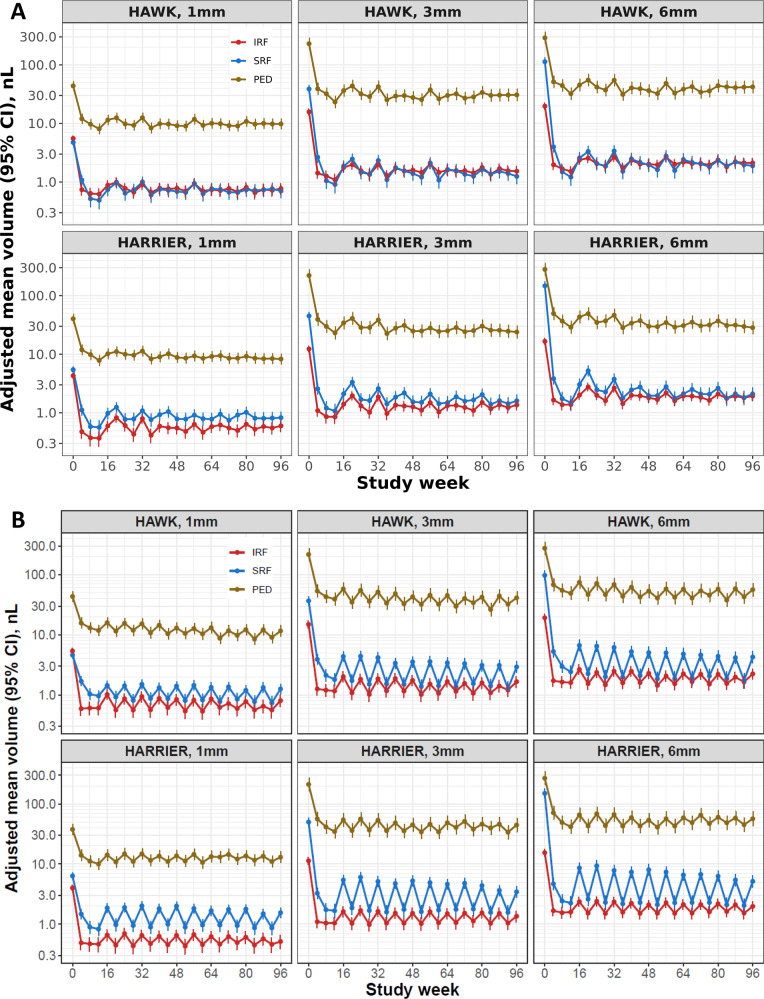
Adjusted mean fluid volumes. **A** Brolucizumab 6 mg and **B** aflibercept 2 mg. Fluid volumes are presented over the 96-week follow-up in the central 1 mm, 3 mm and 6 mm macular fields for HAWK (upper row) and HARRIER (lower row). Error bars denote 95% CIs. CI confidence interval, IRF intraretinal fluid, SRF subretinal fluid, PED pigment epithelial detachment.

The result of modelling the adjusted mean volumes in all three fluid compartments, for the central 1, 3 and 6 mm for brolucizumab 6 mg and aflibercept 2 mg treatment in both studies is shown in Fig. 1. IRF was mainly located in the central 1 and 3 mm macular areas. In both studies, IRF resolved by 92–94% from baseline following the first intravitreal injection and remained as low as a mean 1nL in the central 1 mm during the entire duration of the studies. SRF volume quantities at baseline were 5–10 times higher than IRF volumes. In addition, SRF extended further out than IRF into the 3 and 6 mm macular areas. SRF volumes decreased from baseline by 74–97% following the first injection. PED volumes were initially higher in quantity than SRF volumes and similarly to IRF mostly limited to the central 3 mm macular field. The mean PED volume decreased by 68–79% from its baseline value during initial loading, but thereafter remained at this level during the entire maintenance phase. The results for brolucizumab 3 mg for HAWK is shown in Fig. S2.

**Fig. 2:**
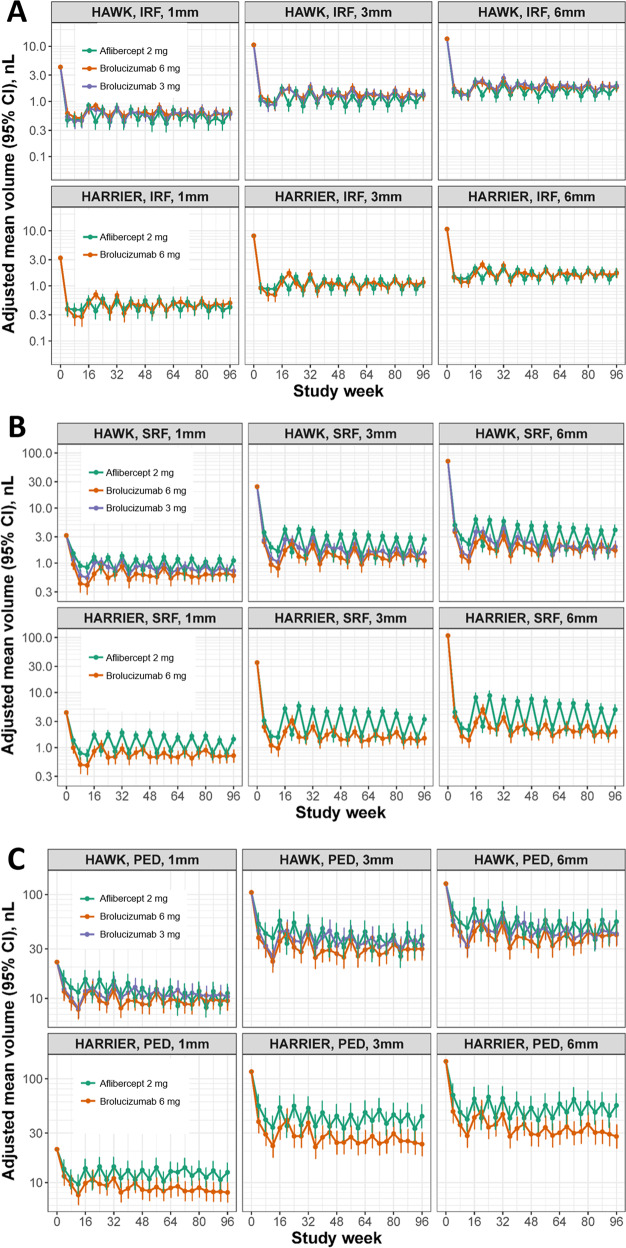
Adjusted mean fluid volumes. **A** Intraretinal fluid, **B** subretinal fluid, and pigment epithelium detachment **C**. Fluid volumes are presented over the 96-week follow-up in the central 1 mm, 3 mm, and 6 mm macular fields for HAWK (upper row) and HARRIER (lower row). Error bars denote 95% CIs. CI confidence interval, IRF intraretinal fluid, SRF subretinal fluid, PED pigment epithelial detachment.

For all treatment arms, the result of modelling of the adjusted mean volumes in all three compartments, in the central 1, 3 and 6 mm, is shown in Fig. 2. With respect to IRF resolution, there were no statistically significant differences between the study arms as both anti-VEGF substances were highly effective in resolving IRF. The saw tooth pattern seen with q8w aflibercept in IRF volumes remained within 1nL, and fluctuations confounded by the asynchronous q8w and q12w regimens across patients in the brolucizumab arms also ranged within <1nL. SRF resolution with brolucizumab was greater under an identical injection schedule up to Week 16 and this advantage was maintained throughout the study duration despite the more frequent retreatments with aflibercept. Statistical analysis showed a superior SRF volume resolution with brolucizumab 6 mg compared to aflibercept 2 mg (*p* < 0.001 for the difference in mean post-baseline SRF) in both studies. This trend was consistent across all macular fields. The fluctuations in SRF volumes in the aflibercept arm were more pronounced than those for IRF in the same group, ranging from a variability of ~1nL in the central 1 mm to ~6nL in the 6 mm area. Similar to SRF, PED resolution with brolucizumab was greater under an identical injection schedule up to week 16 and was maintained throughout the study duration. Statistical analysis revealed that in the central 6 mm the mean post-baseline PED volume obtained with brolucizumab 6 mg was significantly lower than the one obtained with aflibercept 2 mg in HARRIER (*p* = 0.005), with the same trend in HAWK (*p* = 0.15). This trend was found consistently across all macular fields.

The results of modelling the adjusted mean volumes of the three compartments for specific patient subgroups are provided in the supplement. Analyses of the subgroups defined by OCT device (Cirrus, Spectralis, Topcon) are shown in Figs. S3–S5, and show marginally larger IRF volumes being measured with a Cirrus device than with Spectralis, likely due to its larger image resolution (128 B-scans vs. 49 B-scans). Analyses of the subgroups defined by age (<75, ≥75 years) are shown in Fig. S6–S8, where the lower age subgroup exhibited marginally larger SRF volumes. Finally, analyses of the subgroups defined by ethnicity (Asian/Caucasian/other) are shown in Figs. S9–S11, where PED volumes in Caucasians were markedly higher than PED volumes in Asians, likely due to a different MNV subtype distribution between the two cohorts.

### Correlation of fluid volumes and BCVA response

**Fig. 3:**
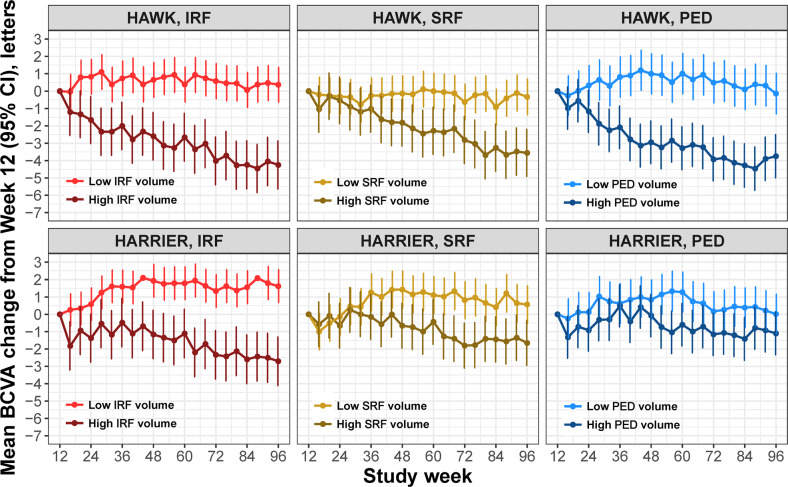
BCVA change for *Low* and *High* fluid volumes. Adjusted mean BCVA change from week 12 for patient subgroups characterised by *Low* or *High* fluid volume in the central 6 mm in each of the three fluid compartments (IRF, SRF, and PED). Error bars denote 95% CIs. BCVA best corrected visual acuity, CI confidence interval, IRF intraretinal fluid, PED pigment epithelial detachment, SRF subretinal fluid.

**Fig. 4:**
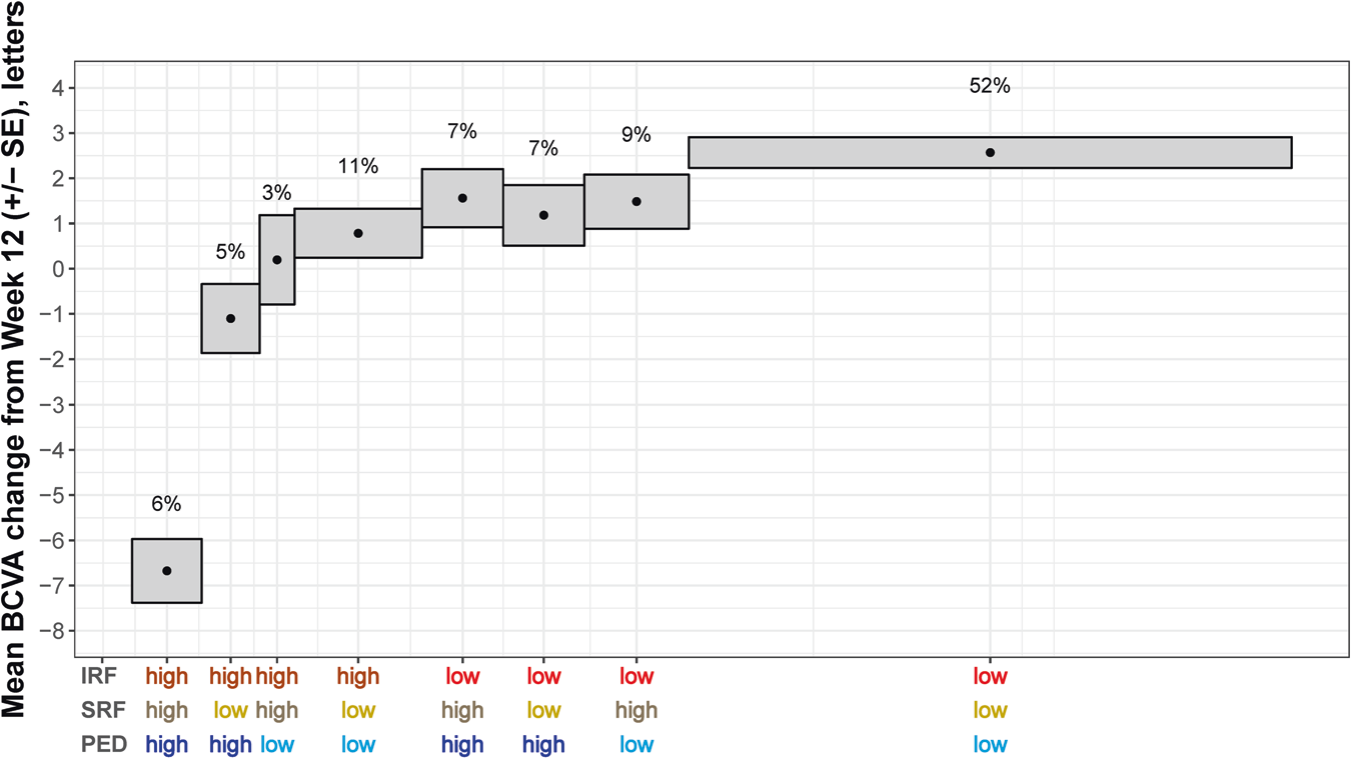
Mean BCVA change from week 12 until the end of the study. The results are averaged over all visits and the two studies. Each box represents a patient subgroup characterised by a different combination of *High/Low* disease activity in each of the three fluid compartments (IRF, SRF and PED). Height of the boxes represents standard error; width of the boxes is proportional to the percentage of patients in the corresponding subgroup. BCVA best corrected visual acuity, IRF intraretinal fluid, PED pigment epithelial detachment, SE standard error, SRF subretinal fluid.

The results of modelling the adjusted mean BCVA change over time from week 12 for each fluid compartment in the central 6 mm individually for *High/Low* volume subgroups are shown in Fig. 3, providing a treatment-agnostic correlation of fluid volumes and BCVA over the maintenance phase. We observed that lower levels of any fluid (IRF, SRF or PED) volume were associated with better visual outcomes over time when adjusted for the other fluid compartments. Between the *High* and *Low* volume subgroups, the difference in mean BCVA change were 3.4 letters for IRF (*p* < 0.0001), around 1.7 letters for SRF (*p* < 0.001) and 2.5 letters for PED (*p* < 0.0001), averaged over both studies and all time points. This finding revealed that in addition to IRF, larger residual SRF and PED volumes were also associated with progressive vision loss during follow-up. Finally, averaging the fluid volume/function correlation over both studies and all visits, we evaluated the mean BCVA change from week 12 for all combinations of fluid volumes thus defining subgroups of differential disease activity (Fig. 4). The largest BCVA benefit with a mean of +2.5 letters (*p* < 0.001) was observed in the subgroup characterised by *Low* disease activity in all compartments. In contrast, a most substantial visual loss with a mean of −6.5 letters (*p* < 0.001) was observed in subgroups with *High* disease activity in all three fluid compartments. Combinations of different high and low volumes ranged in between these two boundaries.

## Discussion

In contrast to human expert procedures in clinical practice, AI tools can automatically and precisely quantify fluid volumes in different compartments to measure disease activity and therapeutic efficacy. Automated quantification of fluid using AI therefore offers an ideal tool to determine therapeutic efficacy in an objective and reproducible manner, and to help improve the management of nAMD in both clinical trial and real-world settings. In our study, AI-based analysis of fluid volumes from a large set of 41,840 volumetric SD-OCT scans allowed for an individualised yet global conclusion of fluid amounts and function, and confirmed that fluid in all compartments mattered regarding visual outcomes. Lower levels of IRF, SRF and PED during maintenance were all associated with superior visual outcomes. Thus, fluid volumes in all three fluid compartments must be taken into account as markers of disease activity equally and independently relevant for visual function in nAMD, which has major implications for treatment decisions.

The accurate quantitative analysis of the therapeutic fluid response based on realistic volumes reveals characteristic mechanisms of action with different substance features. In respect to brolucizumab, this feature design comprises its structure with a lack of fragment crystallisable domains improving bioavailability and small size adding better target-tissue penetration [17, 18]. Simultaneously, the anti-VEGF binding capacity of brolucizumab is about 22 times greater than that of aflibercept and should also more easily reach RPE and choroidal layers.

Brolucizumab as an innovative intravitreal therapeutic biologic format can come at the costs of higher risks, comprehensively discussed in the recent literature. Since first regulatory approval of brolucizumab in September 2019, treating ophthalmologists started to report incidences of vasculitis including occlusive retinal vasculitis (Beovu update for ASRS members). From these reports, the ASRS reviewed clinical data from 25 eyes including 23 eyes with imaging (ASRS Beovu update). 92% of cases were associated with inflammation and 84% of vasculopathy events were reported as occlusive with arterial vessels most commonly affected (Beovu update ASRS). The ReST committee therefore recommended a careful evaluation for any signs of active inflammation prior to brolucizumab administration as brolucizumab and aflibercept are contraindicated per FDA labels in the setting of inflammation. Novartis appointed an independent Safety Review Committee in March 2020 to independently review these post-marketing cases as well as to perform an unmasked post-hoc review of all cases of investigator-reported intraocular inflammation, retinal vascular occlusions and endophthalmitis in HAWK an HARRIER. (SRC communication, June 2020). The review of the HAWK and HARRIER cases revealed occlusive vasculitis in 2.1% of patients with 1 in 200 individuals beginning brolucizumab therapy losing six or more lines in vision in association with occlusive vasculitis, at the end of study participation. Based on the review of the post-marketing cases, these events have also been added to the Beovu^®^ label.

Association between fluid and function in nAMD is highly complex and non-linear as the disease is also associated with progressive alteration of neurosensory layers such as the outer nuclear layer, the photoreceptor and the RPE layers. This is consistent with the lack of correlation observed between CRT and BCVA. It is by precise quantification of fluid volumes respective to the individual compartments that the role of fluid increase and decrease can be fully understood and demonstrated in a statistically sound manner. Chakravarthy et al have recently highlighted an interdependency of “fluctuation” in fluid volumes and vision loss [19]. Our group has previously demonstrated in an AI-based analysis of the FLUID trials that an increase in residual SRF results in consecutive BCVA loss [20].

Evaluation of fluid defined by different retinal compartments is a relatively novel approach. In clinical trials, retreatment criteria based on retinal fluid are thoroughly defined and overseen by certified experts in a reading centre setting. Nevertheless, manual, i.e. human determination of fluid in and underneath the retina is demanding even for trained individuals. IRF poses the biggest challenge as it is typically presenting with dozens of variable cystoid spaces embedded more or less distinctly within the neurosensory layers. When 1213 pairs of time-domain and SD-OCT scans from the CATT study were analysed by a professional reading centre, agreement on IRF was only 73% with misleading artefactual interpretation of dark areas as IRF [21]. Noteworthy, these discrepancies occurred for a simple qualitative reading of fluid types and not an advanced quantification using deep learning tools. The fact that discordances were seen more commonly with lower total foveal thickness, presence of intraretinal fluid (IRF) only and smaller fluid areas further disqualifies human grading performance during the most sensible maintenance period when volumes are intrinsically low and the neurosensory tissue is progressively becoming atrophic [22]. For detecting retinal fluid in the AREDS 2 study, clinical investigators reached an accuracy of 0.805, a sensitivity of 0.468 and a specificity of 0.970, again demonstrating the limitations of human expert assessment [23]. Although identification of SRF is more reproducible than searching for indistinct cystoid spaces in IRF, measurement of SRF is affected by coexisting features such as subretinal hyperreflective material (SHRM) with borders between SHRM, RPE and SRF difficult to distinguish [24]. IRF and SRF measurements in our AI-based analysis demonstrate consistent and plausible results throughout compartments and localisations including distinct fluctuation patterns resulting from the applied regimen. Regarding SRF specifically, the FLUID study compared outcomes in a prospective design between a SRF-tolerant and a SRF-intolerant regimen and found equal BCVA outcomes in both arms. Yet, as the determination of SRF was performed solely by qualitative reader assessment supplemented by measurement of SRF height, a retrospective deep learning analyses using our fluid algorithm revealed that SRF volumes were in fact similar throughout the study follow-up with no statistical difference in the tolerant or intolerant regimen [25]. Regarding an objective evaluation of retinal fluids, AI-based methods have a vastly superior capacity compared to the human approach.

In the comparative analysis of the HAWK and HARRIER regimen, IRF resolved well with both substances. In general, IRF has been shown to respond rapidly and intensively to anti-VEGF therapy in multiple study settings and different diseases [26–28]. In the primary non-inferiority assessment of visual outcomes, both substances achieved identical functional benefits with a mean change in BCVA from baseline to 96w in HAWK at +5.90 ± 0.78 letters for brolucizumab 6 mg, and +5.3 ± 0.78 letters for aflibercept and in HARRIER + 6.1 letters for brolucizumab 6 mg and +6.6 letters for aflibercept [29]. IRF has recently been suspected to be the strongest driver of visual outcomes. Post-hoc analysis using a qualitative assessment only for presence/absence of IRF in the CATT, EXCITE and VIEW trials revealed that the presence of foveal IRF corresponded to lower BCVA values by up to two lines at baseline and at follow-up in several analyses [30, 31]. Using thorough manual annotation, Waldstein et al showed a tight correlation of IRF and BCVA whereby 60% of baseline BCVA could be explained by three-dimensionally segmented IRF. 40% of the BCVA change by 1 year was explained by changes in IRF-related metrics [30]. In contrast, in the HAWK and HARRIER analyses a fully automated volumetric measurement was performed allowing large scale data analysis and led to a consistent IRF/function correlation which was non-inferior in terms of BCVA gains similar in both substance arms as a consequence of efficient IRF resolution. Interestingly, greater CSFT reductions were observed with brolucizumab 6 mg vs. aflibercept in HAWK and HARRIER. However, objective measurements of IRF demonstrated equal efficacy in IRF resolution consistent with equal BCVA outcomes. This supports the notion of IRF and not CSFT having the strongest impact on vision outcomes, provided that IRF is determined in a reliable manner. Whether the small fluctuations seen in a bimonthly regimen are relevant for BCVA on an individual base, cannot be judged from the averaged overall outcomes. Evans et al extracted foveal centre point thicknesses from CATT and IVAN, and used the concomitant SD for grouping the variations in quartiles with a staggered degree of fluid fluctuation. The investigators found that BCVA worsened significantly across the quartiles with increasing fluctuations with a difference between the first and fourth quartiles representing lowest and highest variations as high as −6.27 letters [32]. This has been confirmed in a retrospective real-world analysis with a loss in visual acuity of 9.5 letters in the fourth quartile [33]. Individualised correlations between BCVA and IRF may reveal such an impact of IRF fluctuations even at the levels of small volume changes documented in our analysis.

The presence of SRF has been reported to show less association with BCVA changes than IRF. In a post-hoc analysis of the CATT population, eyes presenting with foveal SRF at year two demonstrated better BCVA than eyes with extrafoveal or no SRF [34]. In HARBOR post-hoc analyses, SRF and PED ranked low in affecting BCVA values, even inferior to total retinal thickness [11]. In contrast to the consistently negative impact of IRF, SRF correlated positively with BCVA: Per 100nL of IRF, BCVA was reduced by a mean of −4 letters, whereas the same amount of SRF correlated with a mean increase of BCVA by +2 letters [11]. Automated fluid segmentation to quantify fluid volume and function dynamics under therapy and post-baseline, in the HARBOR data revealed that a decrease of IRF and even more so of SRF was reflected in a corresponding increase in BCVA by +2.13 letters per 100nL for decreasing IRF and +5.88 letters per 100nL of resolved SRF [35]. Such correlations represent a convincing guidance for therapeutic regimens indicating that in residual/recurrent SRF patients may benefit from retreatment, even if SRF per se does not infer severe damage to the neurosensory retina, but may represent a missed opportunity, if left untreated. In the FLUID study, Grechenig et al showed that an increase in residual SRF volume following interval extension resulted in a moderate, but statistically significant, negative impact on BCVA [20]. In our volume-based HAWK and HARRIER analyses, fluid in the deeper subretinal layers responded particularly well to brolucizumab therapy confirming the concept of deeper tissue penetration of the smaller and highly concentrated molecules. The saw tooth pattern of aflibercept in comparison is impressive, particularly as aflibercept has been promoted earlier as having a better drying capacity for SRF than ranibizumab as seen after switching substance in patients with persistent fluid [36]. A pooled analysis of 28 studies, however, confirmed that significantly improved anatomical outcomes had no impact on visual function which remained stable after switching [36]. Yet, this lack of functional recovery may result from a long-term damage occurred earlier in these long-term cases and may not be consistent with the effects of timely SRF resolution.

A good efficacy of aflibercept in resolving PED has also been shown in previous studies [37]. Even in refractory PED with or without SRF, PED volumes were significantly reduced from 0.43 mm^3^ at baseline to 0.23 mm^3^ at week 8 using a semi-quantitative manual approach. A precise assessment of PED volumes in the current analyses revealed a superior effect on the reduction of PED using brolucizumab. This capacity may be useful as it demonstrates the impact of the substance on the neovascular lesion origin in MNV types 1 and 2, particularly if durability in reducing the biological disease activity is the goal. The fact that fluctuations at the level of the PED still occur during a follow-up as long as 96 weeks, impressively reflects the progressive nature of disease activity within the subretinal fibrovascular complex.

The most revealing aspect of this paper is the accurate correlation of macular function and fluid resolution enabled by the large dimension and precision of volumetric fluid determination in HAWK & HARRIER. The progressive loss in BCVA during follow-up in the higher volume quartile was clearly confirmed for all fluid types. To set this observation into an adequate proportion with clinical relevance, a modelling of the adjusted mean BCVA change over time from week 12 for each fluid compartment for individual *High/Low* fluid volume subgroups was provided in a treatment agnostic manner. Optimal BCVA outcomes are achieved at low fluid volumes in all categories and higher volumes are associated with the poorest visual function. Nevertheless, larger residual SRF and PED volumes were also associated with vision loss during maintenance. Low IRF in combination with a residual presence of SRF and/or PED is still combined with good BCVA maintenance and the deficit range is limited to two letters. However, resistant IRF at higher levels may still be accompanied by good vision, if SRF and PED volumes have resolved. BCVA declines further, if the PED volume is increasing. A correlation of an expanding PED and increasing IRF has been shown previously as a common mechanism for progressive visual loss in the VIEW studies and may support the value of PED reduction to prevent functional decline due to accumulation IRF [30].

An obvious limitation of this study and the generalisation of our findings is its retrospective nature and the focus on imaging data obtained from an a priori standardised clinical trial setting. AI-based fluid analysis is a novel achievement and has only recently become available for use in large dimensions. Even from large clinical studies only few data sets have so far undergone systematic volumetric fluid quantification [11, 25, 28, 37]. Images from real-world populations are only difficult to assemble due to institutional data protection regulations and the personalised character of OCT features preventing a smooth exchange between institutions [38]. To extrapolate population-based findings to an individual level is always hypothetical which limits our assumptions about BCVA loss or gain induced by IRF/SRF/PED volume changes in a specific individual with nAMD.

In conclusion, automated quantification of retinal fluid volumes based on deep learning in two large randomised controlled trials allowed to evaluate pathognomonic resolution patterns for different retinal compartments and to objectively compare the therapeutic efficacy of pharmacological substances. It is conceivable that such advanced diagnostic methods determining fluid resolution in a reliable quantitative manner, over time and in different disease phenotypes will provide “fingerprint-like“ characteristics for drugs, regimen and modes of administration e.g. implants and strategies such as retinal gene therapy. Moreover, only small advances in clinical outcomes were achieved between different pharmacological developments, yet an accurate therapeutic guidance by an automated and reliable fluid volume measurement may improve the benefit in a more economic and personalised manner. Also, retinal fluid is just one of a multitude of biomarkers which can be analysed and measured by automated algorithms. Many other relevant features are being explored such as hyperreflective foci, photoreceptor integrity, neurosensory layer atrophy etc. Regarding the current anti-VEGF-based therapy of exudative macular disease, a quantitative correlation of fluid volumes with BCVA values offers clinically most relevant insight into the impact of morphological features on neurosensory function as well as prognostic outcomes of intervention. The results of such accurate and objective analyses represent the base of identifying appropriate parameters prospectively guiding disease management in the real world and detecting promising therapeutic targets for innovative treatment strategies in future clinical trials.

## Summary

### What was known before

- Intraretinal fluid is associated with worse visual acuity outcomes in neovascular age-related macular degeneration. Tolerance of SRF might not have an impact on visual acuity outcomes.

### What this study adds

- Eyes with high volumes of IRF, SRF and pigment epithelium detachments have worse visual acuity outcomes compared with eyes with low fluid volumes in the maintenance phase. High fluid volumes in all compartments should be treated to optimise anti-VEGF therapy in neovascular age-related macular degeneration.

## Supplementary information

Supplemental Figure legends

Supplemental Figure 1

Supplemental Figure 2

Supplemental Figure 3

Supplemental Figure 4

Supplemental Figure 5

Supplemental Figure 6

Supplemental Figure 7

Supplemental Figure 8

Supplemental Figure 9

Supplemental Figure 10

Supplemental Figure 11

Supplemental Table 1

## Data Availability

Original data for this research were provided by Novartis Pharma AG. Data that support the findings of this study are available upon reasonable request.
