# Supplementary material for: Therapeutic response in the HAWK and HARRIER trials using deep learning in retinal fluid volume and compartment analysis
Source: Eye (Lond). 2022 May 6;37(6):1160–9. doi: 10.1038/s41433-022-02077-4 (PMC10101971; doi:10.1038/s41433-022-02077-4)
Supplement: Supplementary file 1 — Supplemental Figure legends [file 41433_2022_2077_MOESM1_ESM.docx]

**Figure S1.** Illustration of the HAWK and HARRIER prospective phase 3 clinical trial designs. In HAWK, disease activity assessments were conducted by masked investigators at week 16, week 20 and every 12 weeks thereafter to determine the subsequent dosing interval. There were additional disease activity assessment visits in the HARRIER trial. q8w, 8-week dosing interval; q12w, 12-week dosing interval.

**Figure S2**. Adjusted mean fluid volumes for brolucizumab 3mg over the 96-week in the central 1 mm, 3 mm and 6 mm macular fields for HAWK. Error bars denote 95% CIs. CI, confidence interval; IRF, intraretinal fluid; SRF, subretinal fluid, PED, pigment epithelial detachment.

**Figure S3**. Adjusted mean **IRF** volumes pooled for each **OCT device**, over the 96-week in the central 1 mm, 3 mm and 6 mm macular fields for HAWK (upper row) and HARRIER (lower row). Error bars denote 95% CIs. CI, confidence interval; IRF, intraretinal fluid.

**Figure S4**. Adjusted mean **SRF** volumes pooled for each **OCT device**, over the 96-week in the central 1 mm, 3 mm and 6 mm macular fields for HAWK (upper row) and HARRIER (lower row). Error bars denote 95% CIs. CI, confidence interval; SRF, subretinal fluid.

**Figure S5**. Adjusted mean **PED** volumes pooled for each **OCT device**, over the 96-week in the central 1 mm, 3 mm and 6 mm macular fields for HAWK (upper row) and HARRIER (lower row). Error bars denote 95% CIs. CI, confidence interval; PED, pigment epithelial detachment.

**Figure S6**. Adjusted mean **IRF** volumes pooled for each **racial group**, over the 96-week in the central 1 mm, 3 mm and 6 mm macular fields for HAWK (upper row) and HARRIER (lower row). Error bars denote 95% CIs. CI, confidence interval; IRF, intraretinal fluid.

**Figure S7**. Adjusted mean **SRF** volumes pooled for each **racial group**, over the 96-week in the central 1 mm, 3 mm and 6 mm macular fields for HAWK (upper row) and HARRIER (lower row). Error bars denote 95% CIs. CI, confidence interval; SRF, subretinal fluid.

**Figure S8**. Adjusted mean **PED** volumes pooled for each **racial group**, over the 96-week in the central 1 mm, 3 mm and 6 mm macular fields for HAWK (upper row) and HARRIER (lower row). Error bars denote 95% CIs. CI, confidence interval; PED, pigment epithelial detachment.

**Figure S9**. Adjusted mean **IRF** volumes pooled for each **age group**, over the 96-week in the central 1 mm, 3 mm and 6 mm macular fields for HAWK (upper row) and HARRIER (lower row). Error bars denote 95% CIs. CI, confidence interval; IRF, intraretinal fluid.

**Figure S10**. Adjusted mean **SRF** volumes pooled for each **age group**, over the 96-week in the central 1 mm, 3 mm and 6 mm macular fields for HAWK (upper row) and HARRIER (lower row). Error bars denote 95% CIs. CI, confidence interval; SRF, subretinal fluid.

**Figure S11**. Adjusted mean **PED** volumes pooled for each **age group**, over the 96-week in the central 1 mm, 3 mm and 6 mm macular fields for HAWK (upper row) and HARRIER (lower row). Error bars denote 95% CIs. CI, confidence interval; PED, pigment epithelial detachment.
