# Supplementary figures and images for: Therapeutic response in the HAWK and HARRIER trials using deep learning in retinal fluid volume and compartment analysis
Source: Eye (Lond). 2022 May 6;37(6):1160–9. doi: 10.1038/s41433-022-02077-4 (PMC10101971; doi:10.1038/s41433-022-02077-4)

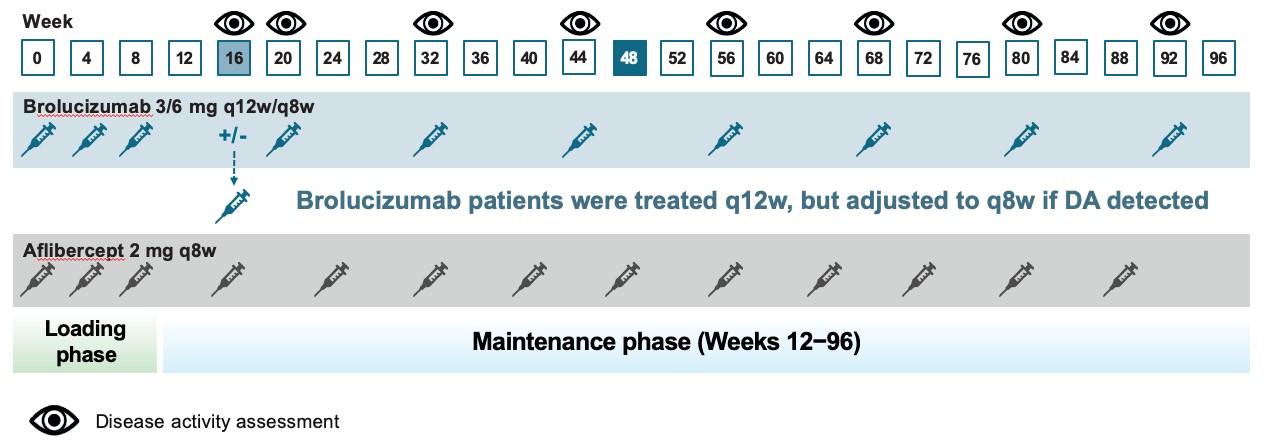

Supplement: Supplementary file 2 — Supplemental Figure 1 [file 41433_2022_2077_MOESM2_ESM.jpg]

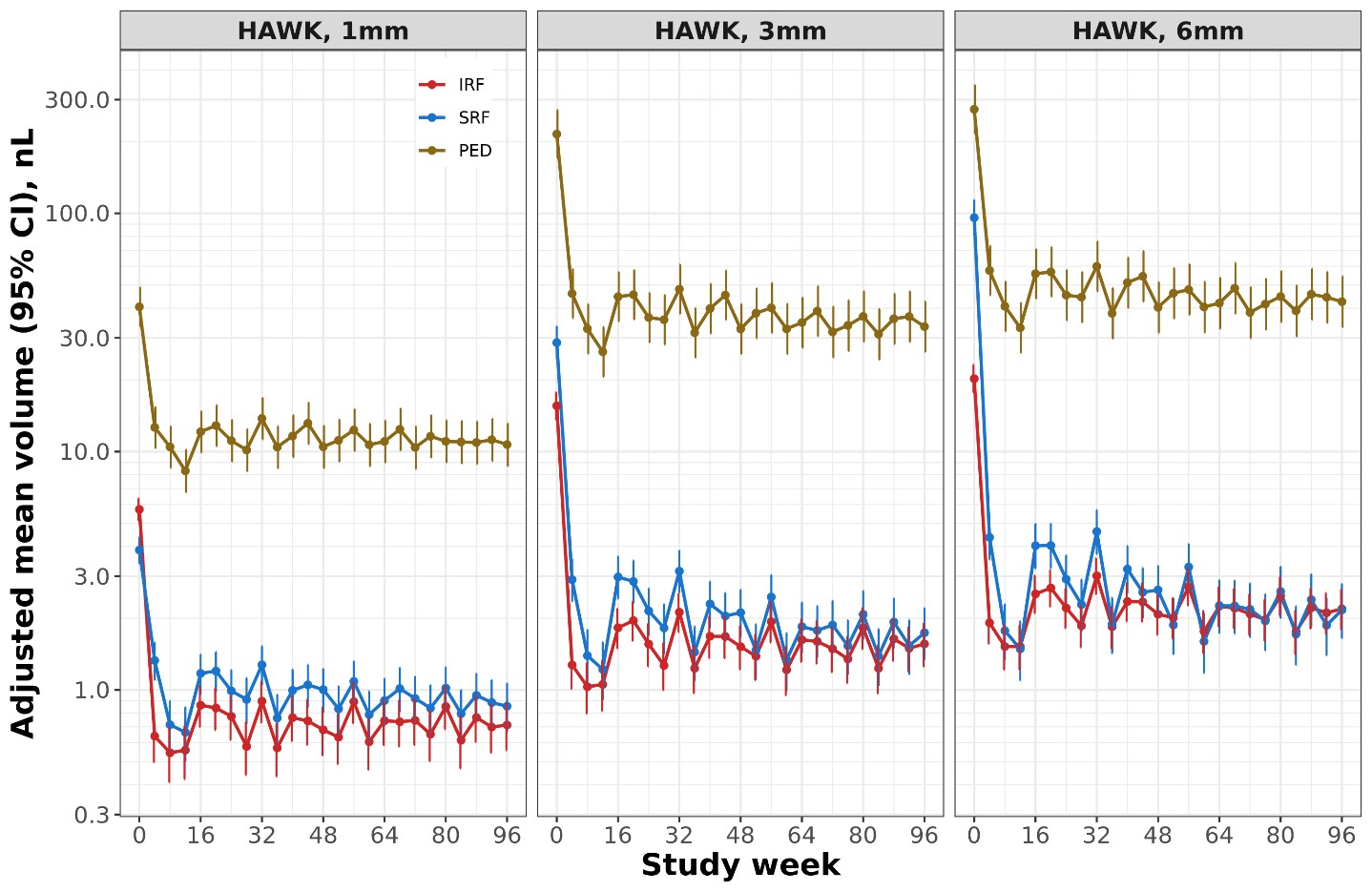

Supplement: Supplementary file 3 — Supplemental Figure 2 [file 41433_2022_2077_MOESM3_ESM.jpg]

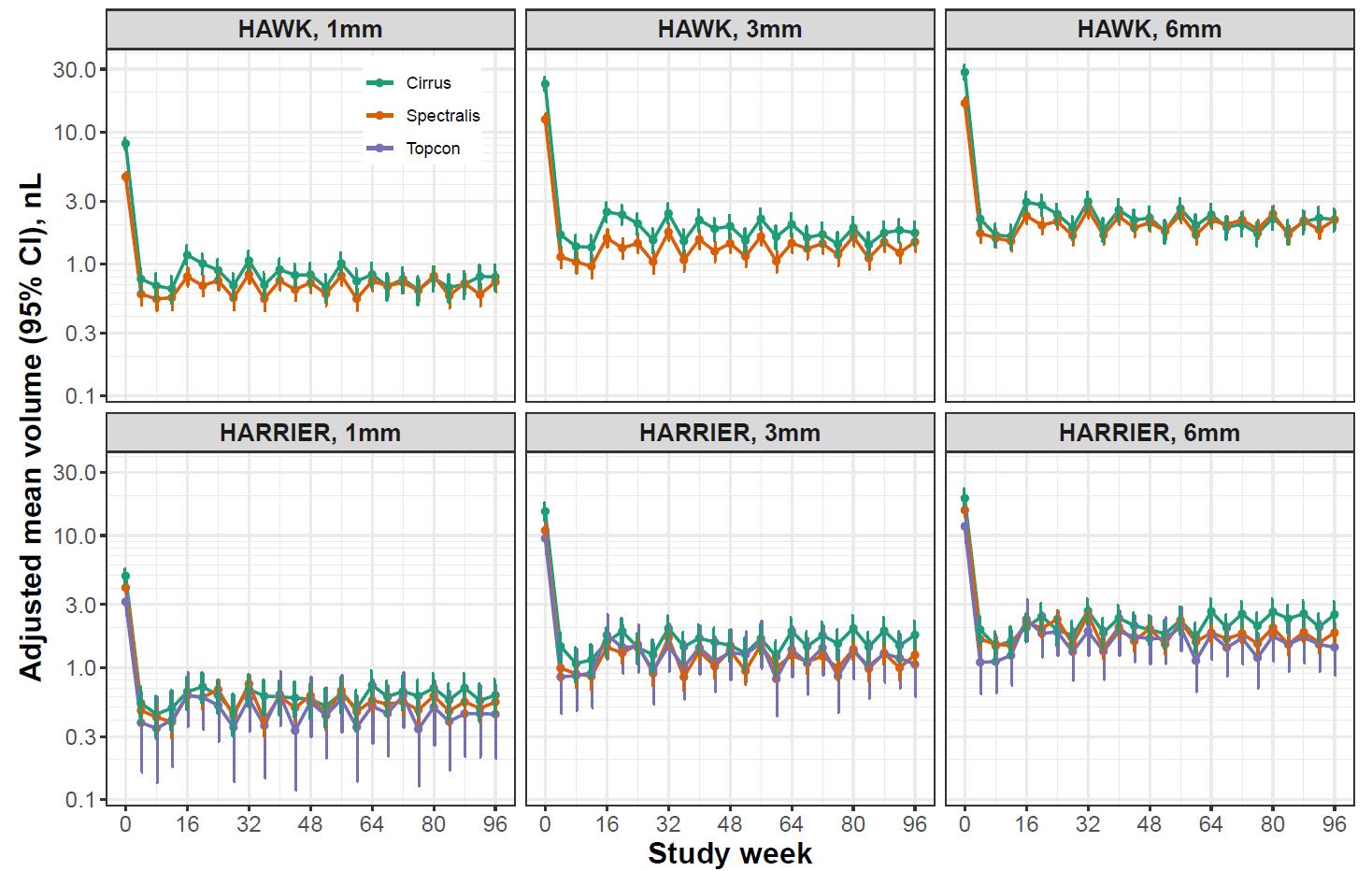

Supplement: Supplementary file 4 — Supplemental Figure 3 [file 41433_2022_2077_MOESM4_ESM.jpg]

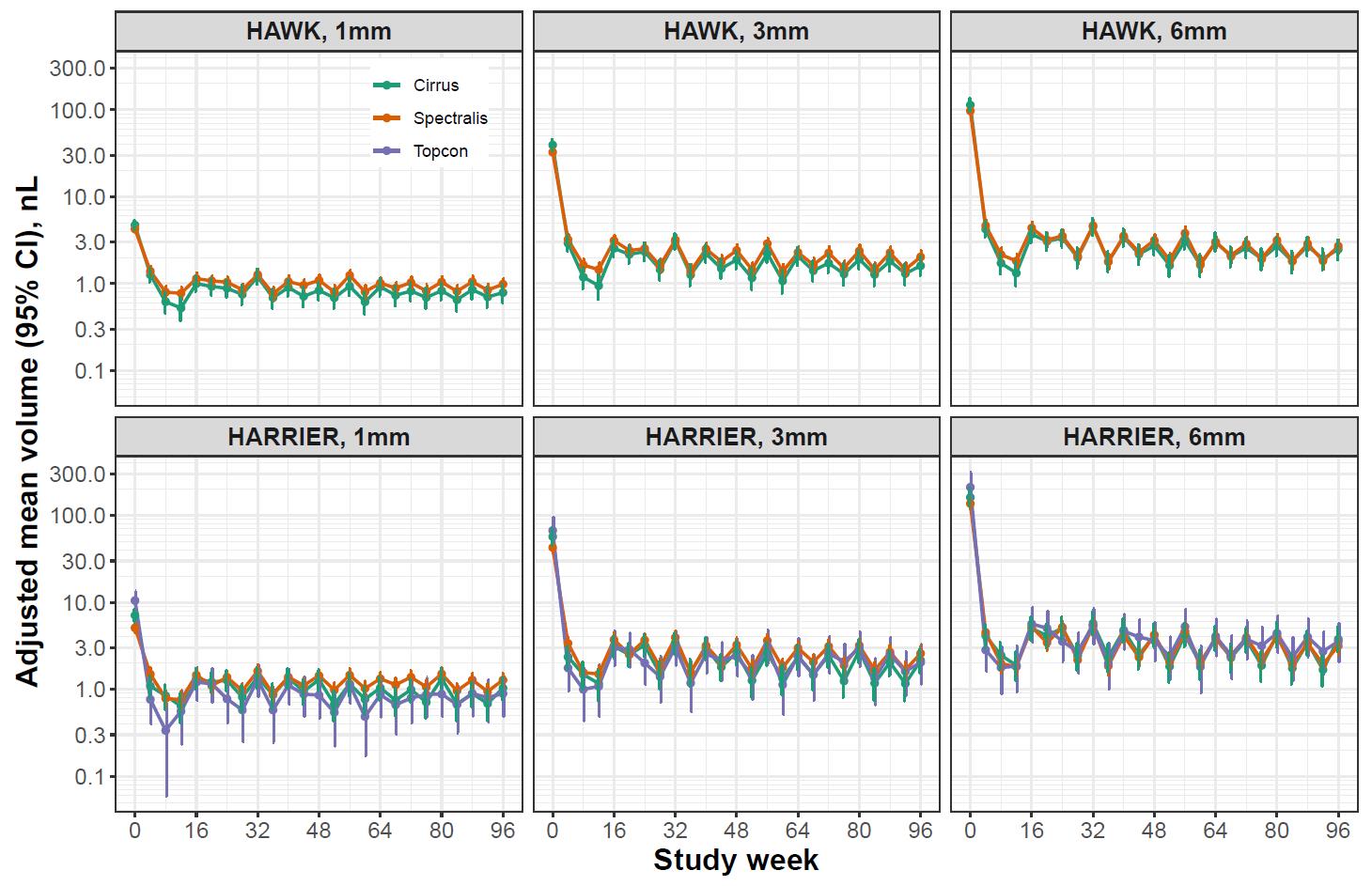

Supplement: Supplementary file 5 — Supplemental Figure 4 [file 41433_2022_2077_MOESM5_ESM.jpg]

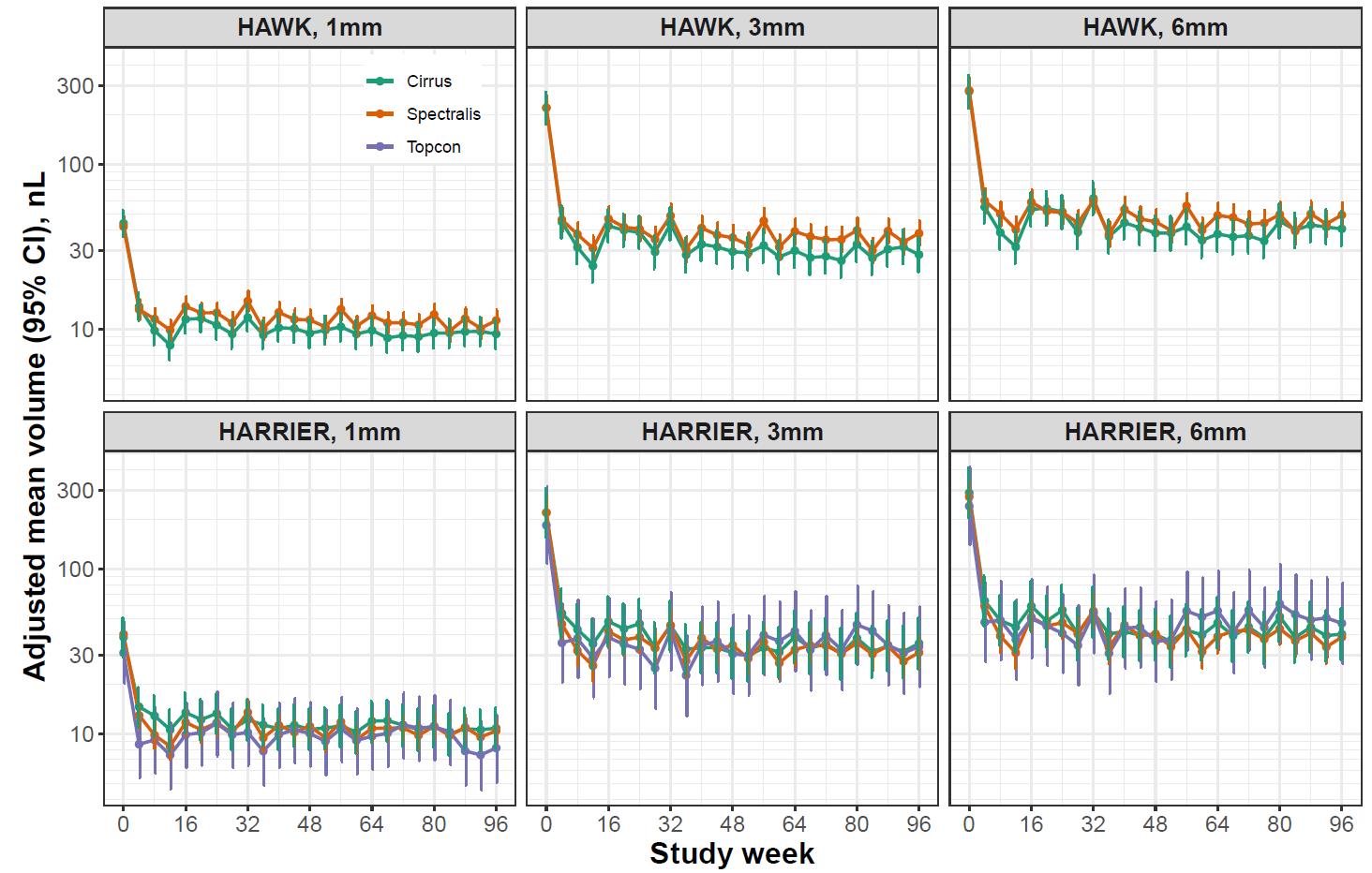

Supplement: Supplementary file 6 — Supplemental Figure 5 [file 41433_2022_2077_MOESM6_ESM.jpg]

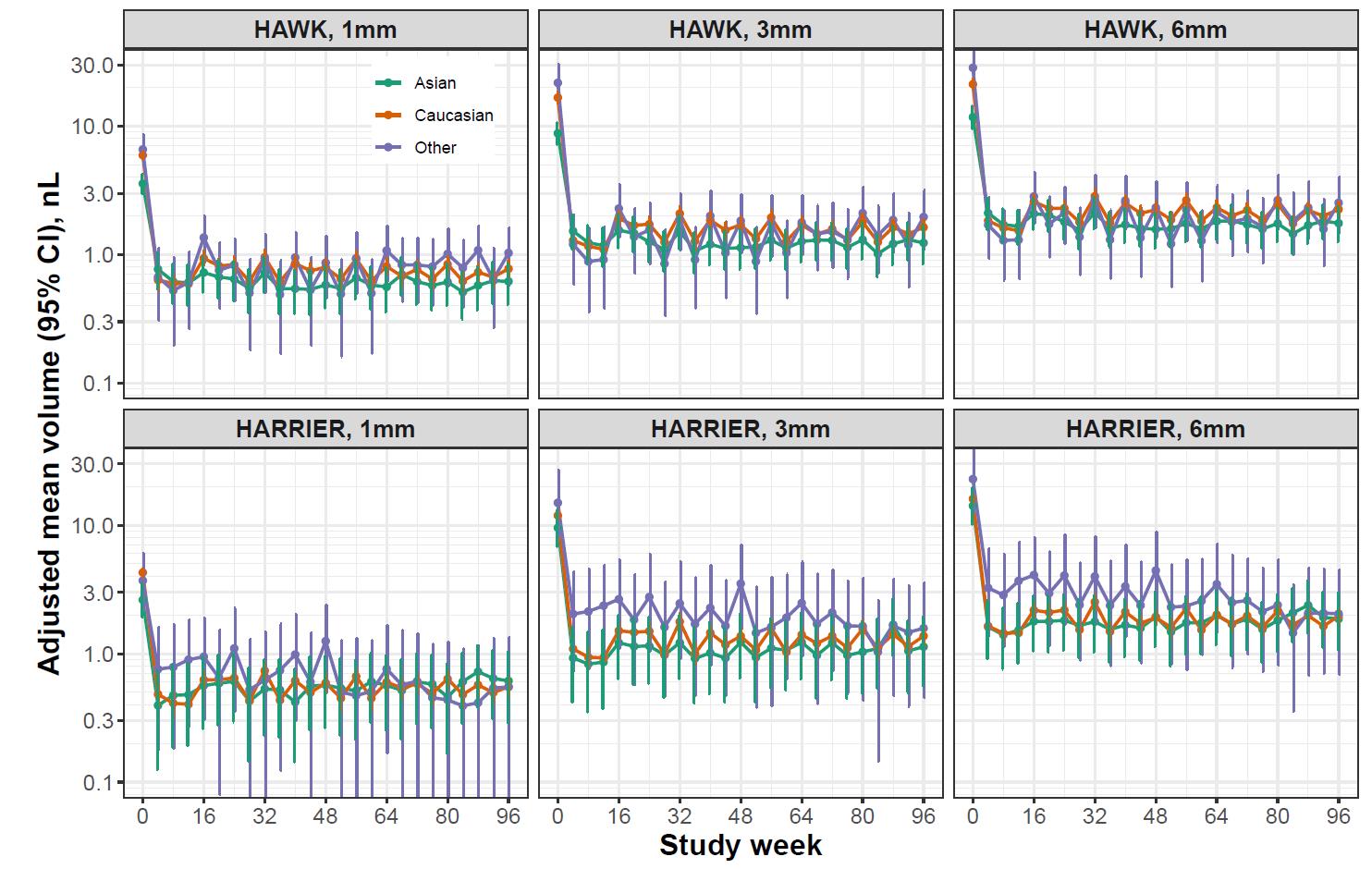

Supplement: Supplementary file 7 — Supplemental Figure 6 [file 41433_2022_2077_MOESM7_ESM.jpg]

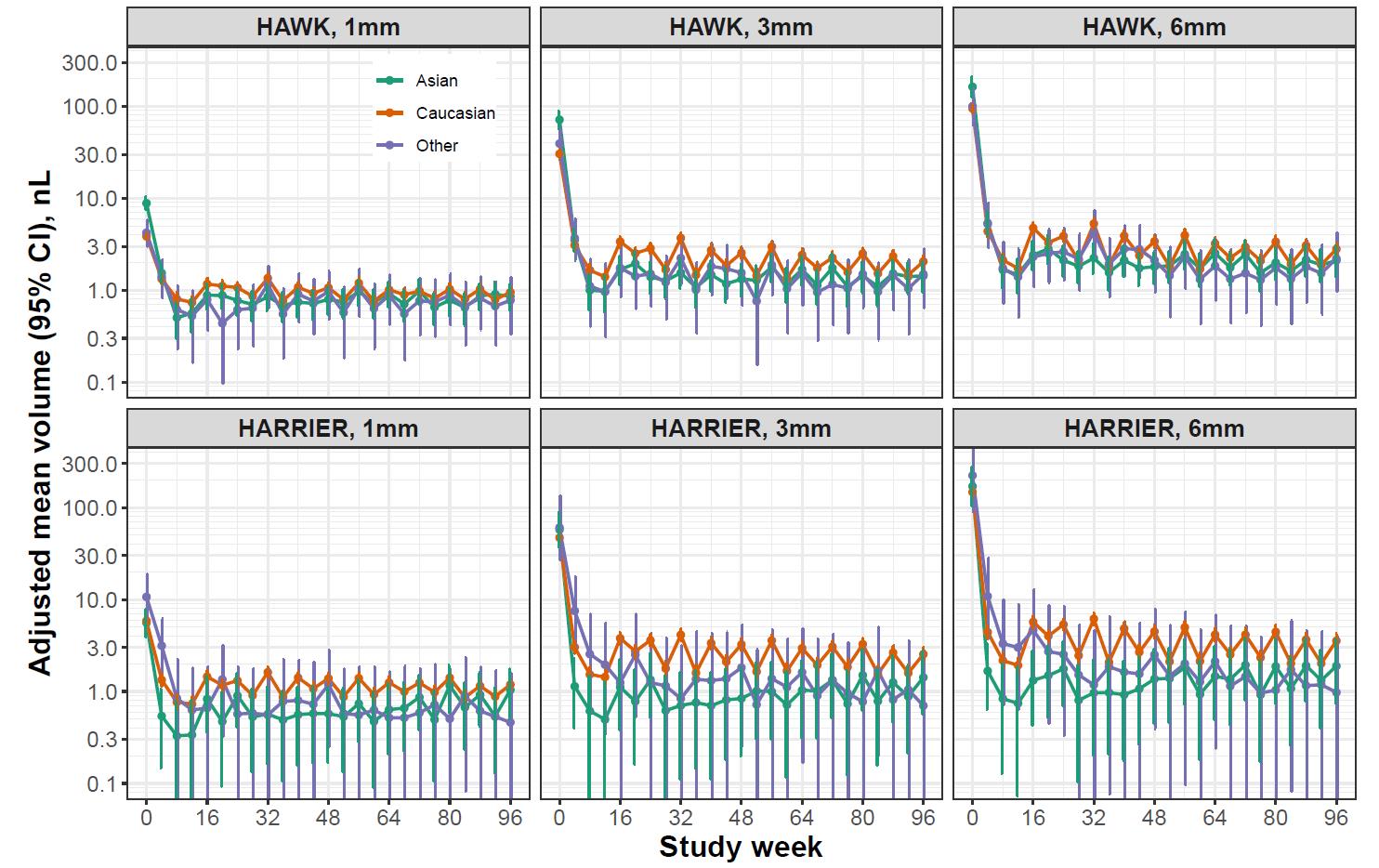

Supplement: Supplementary file 8 — Supplemental Figure 7 [file 41433_2022_2077_MOESM8_ESM.jpg]

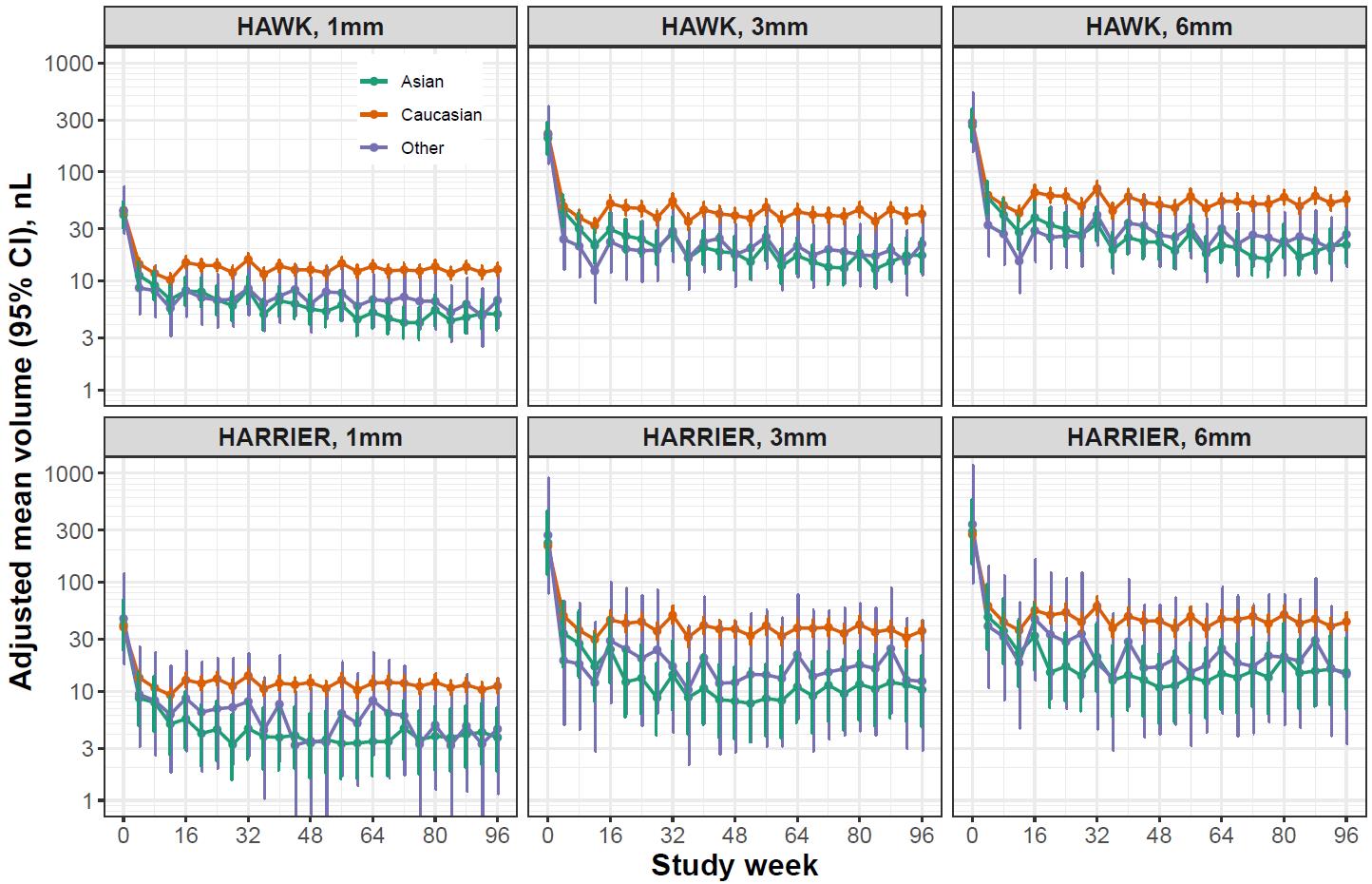

Supplement: Supplementary file 9 — Supplemental Figure 8 [file 41433_2022_2077_MOESM9_ESM.jpg]

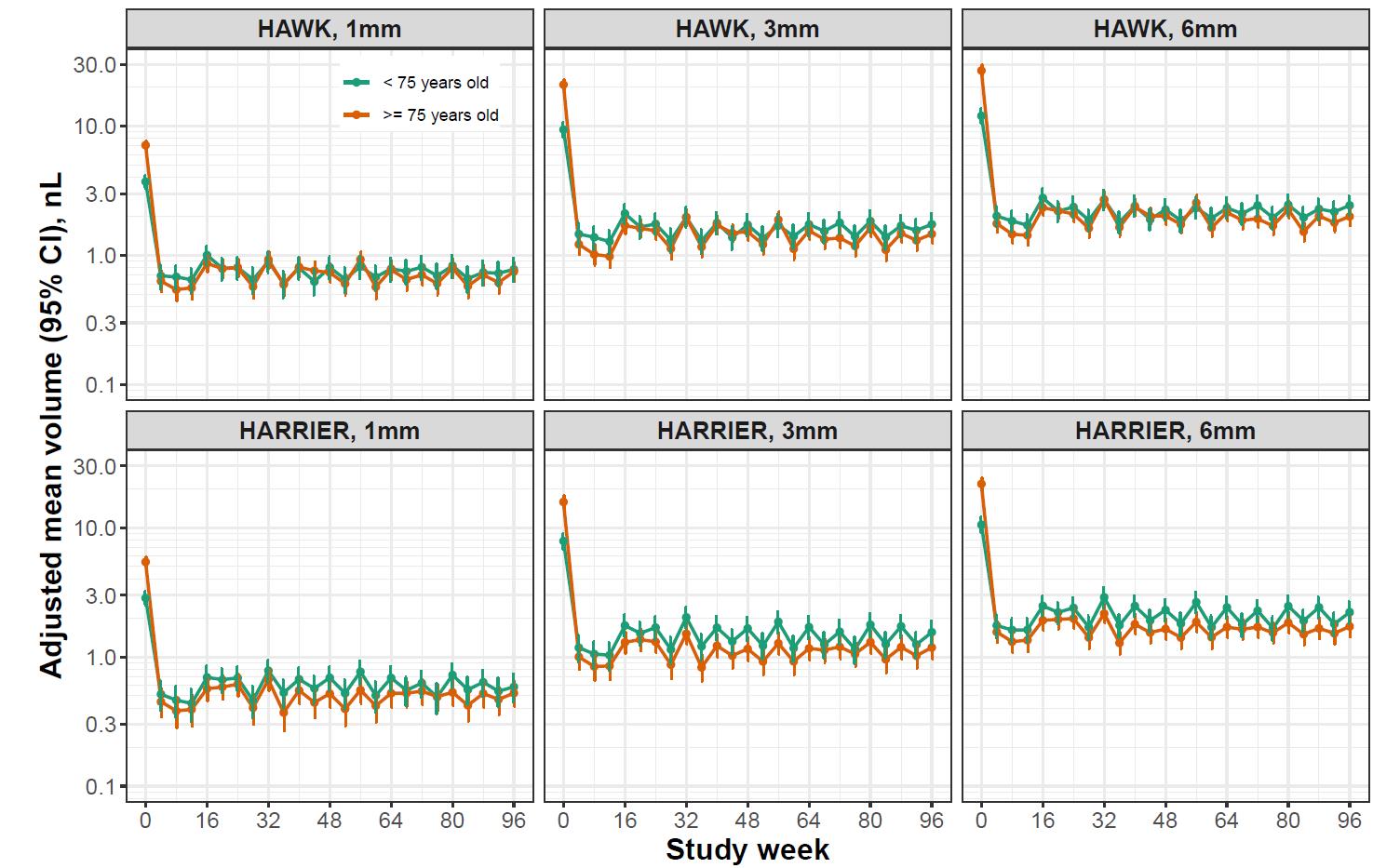

Supplement: Supplementary file 10 — Supplemental Figure 9 [file 41433_2022_2077_MOESM10_ESM.jpg]

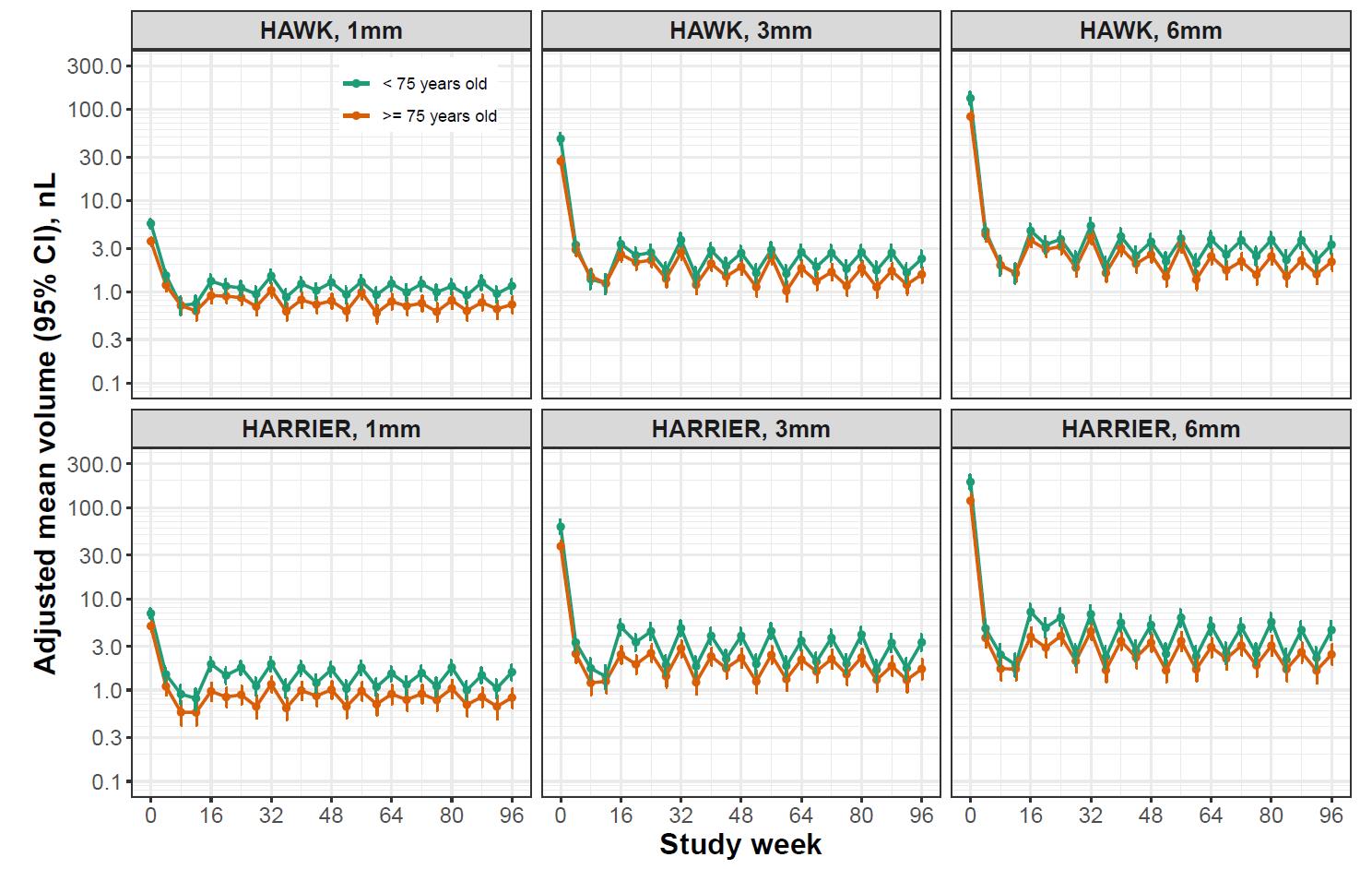

Supplement: Supplementary file 11 — Supplemental Figure 10 [file 41433_2022_2077_MOESM11_ESM.jpg]

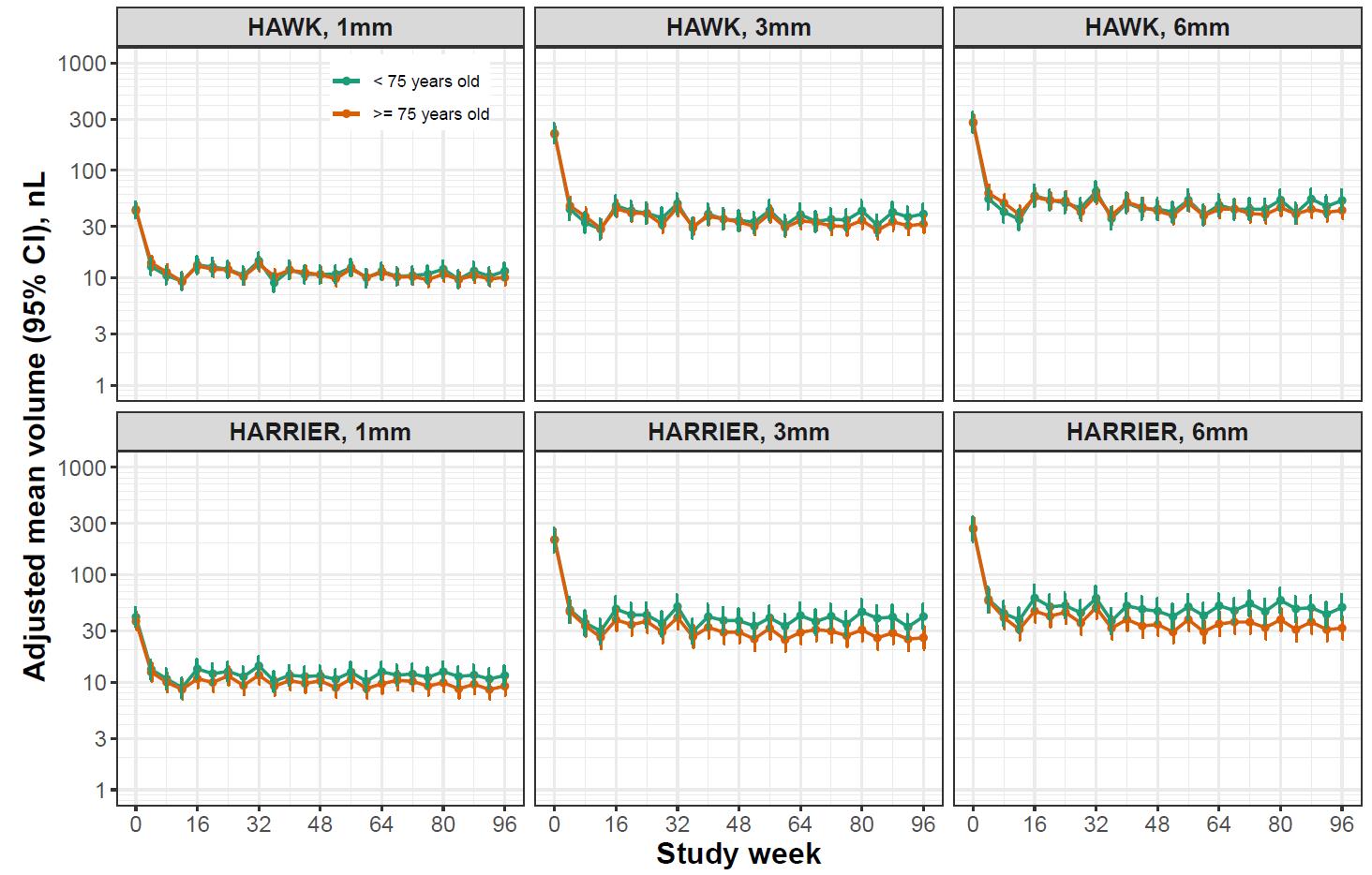

Supplement: Supplementary file 12 — Supplemental Figure 11 [file 41433_2022_2077_MOESM12_ESM.jpg]
