# Supplementary material for: Therapeutic response in the HAWK and HARRIER trials using deep learning in retinal fluid volume and compartment analysis
Source: Eye (Lond). 2022 May 6;37(6):1160–9. doi: 10.1038/s41433-022-02077-4 (PMC10101971; doi:10.1038/s41433-022-02077-4)
Supplement: Supplementary file 13 — Supplemental Table 1 [file 41433_2022_2077_MOESM13_ESM.docx]

**Table S1.** Breakdown of the study by the OCT device used.

| **Study** | **Drug Type** | **Dosage** | **OCT** | **#patients** | **#scans** |
| --- | --- | --- | --- | --- | --- |
| HAWK | Aflibercept | 2mg | Cirrus | 120 | 2505 |
| HAWK | Aflibercept | 2mg | Spectralis | 250 | 5385 |
| HAWK | Brolucizumab | 6mg | Cirrus | 125 | 2737 |
| HAWK | Brolucizumab | 6mg | Spectralis | 241 | 5289 |
| HAWK | Brolucizumab | 3mg | Cirrus | 111 | 2461 |
| HAWK | Brolucizumab | 3mg | Spectralis | 249 | 5603 |
| HARRIER | Aflibercept | 2mg | Cirrus | 96 | 2257 |
| HARRIER | Aflibercept | 2mg | Spectralis | 236 | 5429 |
| HARRIER | Aflibercept | 2mg | Topcon | 35 | 838 |
| HARRIER | Brolucizumab | 6mg | Cirrus | 82 | 1939 |
| HARRIER | Brolucizumab | 6mg | Spectralis | 250 | 5872 |
| HARRIER | Brolucizumab | 6mg | Topcon | 37 | 880 |
